# Effects of hot air treatment and chitosan coating on citric acid metabolism in ponkan fruit during cold storage

**DOI:** 10.1371/journal.pone.0206585

**Published:** 2018-11-16

**Authors:** Yang Gao, Chaonan Kan, Chunpeng Wan, Chuying Chen, Ming Chen, Jinyin Chen

**Affiliations:** 1 Jiangxi Key Laboratory for Postharvest Technology and Nondestructive Testing of Fruits & Vegetables, Collaborative Innovation Center of Post-Harvest Key Technology and Quality Safety of Fruits and Vegetables, College of Agronomy, Jiangxi Agricultural University, Nanchang, China; 2 Pingxiang University, Pingxiang, China; Huazhong Agriculture University, CHINA

## Abstract

In citrus fruit, citric acid is the predominant organic acid which influence fruit taste, flavor and quality. The effect of hot air treatment (HAT 40°C, 48 h) and 1.0% chitosan coating on the change of organic acids and the related gene expression of citric acid synthesis and degradation in ponkan (*Citrus reticulata* Blanco) fruit during cold storage have been studied. The results showed that citric acid was the main organic acid in fruit, the trend change of citric acid content was consistent with total organic acids and titratable acidity (TA) content, which decreased with the prolongation of storage time, hot air treatment significantly promoted but chitosan coating treatment significantly delayed citric acid degradation in Ponkan fruit. Hot air treatment could induced *CitAco2/3*, *CitIDH2/3*, Cit*GAD4*, *CitACLs*, *CitPEPCKs* and *CitFBPases* expression during fruit storage period, but had no significant effect on *CitGSs* expression, The enhanced expression of degradation-related genes was closely related to the degradation of citric acid. The expressions of *CitAco3*, *CitGAD4* Cit*ACLα2*/*β*, *CitPEPCKs* and *CitFBPases* were inhibited, which leading to the degradation rate of citric acid was slowed by chitosan coating during storage. These results showed that the degradation of citric acid in fruit was regulated by ATP citrate lyase (ACL) pathway and γ-aminobutyric acid (GABA) pathway.

## Introduction

Acid is an important indicator of fruit quality. Depending on the type of major organic in ripe fruit, the fruit can be divided into three types: citric acid type, malic acid type and tartaric acid type fruit. Citrus fruit is citric acid type fruit Main text paragraph [1]. Ponkan (*Citrus reticulata* Blanco) is one of the main mandarin citrus in China, the fruit are rich of organic acid [2]. Zhang et al. [3] have demonstrated that citric acid was the major organic acid in Ponkan fruit, which accountings for 91.82% of the total content in single fruit. During the storage period, the content of organic acids in fruit not only affected fruit flavor and quality, but also affected the storage characteristics of fruit [4].

A large number of studies have shown that the appropriate postharvest treatment could effectively affect the fruit organic acid content, improve fruit storage quality. As a viable alternative to synthetic fungicides, the clove extract could significantly inhibit the water loss rate and decay rate in ‘Newhall’ navel orange fruit during storage, delay the degradation of TA, TSS and V_C_, and improve antioxidant enzymes activity, then the fruit could maintain good quality at the later stage of storage [5]. 1-methylcyclopropene treatment could maintain the apple acidity during fruit storage by adjusting the balance between malic acid biosynthesis and degradation [6]. Exogenous GABA treatment could decrease the fruit rot rate, increase the content of organic acids, maintenance and improvement of the storage performance of citrus fruits [7].

Hot air treatment as a common method has been used for many species of fruit and vegetable after harvest, such as strawberry [8], peaches [9], 'Qingnai' plum [10] and citrus fruit [2, 11]. Heat treatment of 50°C for 5–10 minutes could delay the change of storage quality of sweet orange fruit and reduce the occurrence of fruit decay [12]. Hot air treatment could enhance oxygen scavenging and cell wall polysaccharides solubilization, thus enhancing the cold resistance of loquat fruits [13]. Chitosan, is a natural polysaccharide from a wide range of sources [14]. Due to its biocompatibility, biodegradability, bacteriostasis and film formation, chitosan has great potential in various fields [15–17]. Chitosan coating treatment, as a postharvest preservation method, has also been studied for many fruit [18, 19]. The effect of chitosan coating on the antioxidant activity and malic acid metabolism were studied in ‘Huangguan’ pear during storage, the results showed that chitosan treatment had a significantly positive effect on the activities of NAD-MDH and a negative effect on the activities of NADP-ME both in pulp and peel. It also inhibited the gene expression of *vVAtp2*, as well as promoted gene expression of *vVAtp1* [20]. It was further proved that chitosan coating could effectively reduce the occurrence of fruit anthracnose and postpone the decline of postharvest quality of fruit [21, 22]. After treatment with a range (0.5, 1.0 and 2.0%) of chitosan solutions, respectively. The respiration rate and weight loss of longan fruits were decreased during storage, the increase in PPO activity and the changes in colour were delayed [23].

Most of research were focus on the effects of hot air treatment or chitosan coating on storage of fresh fruit, and their influences on organic acid metabolism during storage stage of citrus fruit were rarely studied. In this study, the changes of organic acids were measured in Ponkan fruit treated by hot air and chitosan coating during cold storage, and the citric acid degradation -related gene expression also were analyzed during storage, then the mechanism of citric acid degradation were discussed in Ponkan fruit during storage.

## Materials and methods

### Ethics statement

The owner of the land gave permission to conduct the study on this site. No specific permissions were required for these locations/activities. And the field studies did not involve endangered or protected species.

### Plant materials

Mature Ponkan (*C*. *reticulata* Blanco cv. Ponkan) fruits were harvested from a commercial orchard in Jing'an county, Jiangxi Province, China (115º 35´E, 28 º 88´N), at the mature stage (21th November, 2015). The fruit were transported to the laboratory for Postharvest Technology and Nondestructive Testing of Fruits & Vegetables, Jiangxi Agriculture University (Nanchang, China) on the day of harvest.

### Treatments and sampling

Fruit in the uniform size and maturity, free of visible disease and mechanical wounding, were divided into 200 fruit per group for postharvest treatments. The fruit were subjected to the following treatments: (1) control: the fruit were directly stored at 10°C, 90–95% RH in a cold room; (2) hot air treatment (HAT): the fruit were placed in a chamber at 40°C, 90–95% RH for 48h, then the fruit were transferred to 10°C with the same conditions as control treatment; (3) chitosan treatment: the fruit were coated with 1.0% chitosan and dried naturally, then the fruit were transferred to 10°C with the same conditions as control treatment. All of the control, HAT and chitosan treatment fruit were stored till 120 d after harvest.

Fruit qualities were measured at 0, 2, 15, 30, 45, 60, 75, 90, 105, 120 d during storage. Each sampling consisted of fifteen fruit for each treatment, separated as three replicates with five fruit each. After measurements, the flesh tissue samples were frozen in liquid nitrogen, and stored at −80°C, for further experiments.

### TA and organic acids measurements

The fifteen fruit were divided into three replicates with five fruit each. Five grams of the filtrated juice for each group was titrated with 0.1 M NaOH to its end point at pH 8.1. The results were expressed as the percentage of citric acid (1g of citric acid per 100 g FW), which represented the titratable acidity (TA) value.

The method of extracted organic acids were modified from the description of Chen. Four grams of frozen flesh sample was ground to a powder in liquid nitrogen, add in 5.0 mL of ethanol (80%) solution and and water bathed at 35°C for 20 min. The extract was centrifuged at 10,000×g for10 min. The residue was extracted twice and the supernatant was collected in volumetric flask to final 25 ml with 80% ethanol. 1 ml extract solution was filtered with Ф0.22 μm, Ф13mm water syringe filter (Shanghai Xingya Purification Material Factory, China). The filtered solution was used for organic acids analysis.

Organic acids were measured using high performance liquid chromatography (HPLC, SHIMADZU LC-20A, Japan). The liquid chromatograph equipped with a degasser, quaternary pump, 20 μL volume injection autosampler, ODS C18 column (4.6 mm×250 mm, Waters Corporation, USA) and a diode array detector. The mobile phase was a solvent system of 50 mM (NH4)_2_HPO4 phosphate buffer (pH = 2.7 adjusted with phosphoric acid) at a flow rate of 0.5 mL/min. Organic acids were detected at a wavelength of 210 nm. Lab Soblutions Software (Shimadzu, Japan) was used to run the HPLC and process the results. Triplicate flesh samples were analyzed.

### RNA extraction and cDNA synthesis

Total RNA was extracted from frozen tissues by CTAB method [24], and the quality was detected by agarose gel electrophoresis, each sample has three biological replicates. Reverse transcription of RNA using reverse transcription kit (TaKaRa's Cat. # RR047A, Japan). The cDNA was used as the template for real-time quantitative PCR analysis.

### Real-time quantitative PCR (Q-PCR) analysis

Q-PCR was performed on a CFX 96 real-time PCR detection system (Bio-Rad, Hercules, CA, USA). The primers used in the qPCR analyses of individual genes were designed according to Chen [25] and Guo [26]. For each q-PCR reaction, 2 μL of each diluted sample was used as atemplate in a 25 μL reaction containing, 12.5 μL of SYBR green supermix (TaKaRa, Japan), 9.5 μL of ddH_2_O and 2 μL of each primer. The following PCR conditions were used: 95°C for 30s, followed by 40 cycles of 95°C for 5 s, 60°C for 30 s, and 95°C for 15 s.

Gene-specific primers were described in previous report [25]. Three different RNA isolations and cDNA syntheses were used as replicates for qRT-PCR.

### Statistical analysis

The experiments were performed using a completely randomized design. Standard errors (SE) and figures were made by GraphPad Prism (GraphPad Software, Inc., 7825 Fay Avenue, LA Jolla, USA). The analysis of significant difference by using the new Duncan type repolarization difference detection method in DPS 7.05 (Zhejiang University, Hangzhou, China).

## Results

### Effect of HAT and chitosan treatment on the content of organic acid and TA

**Fig 1:**
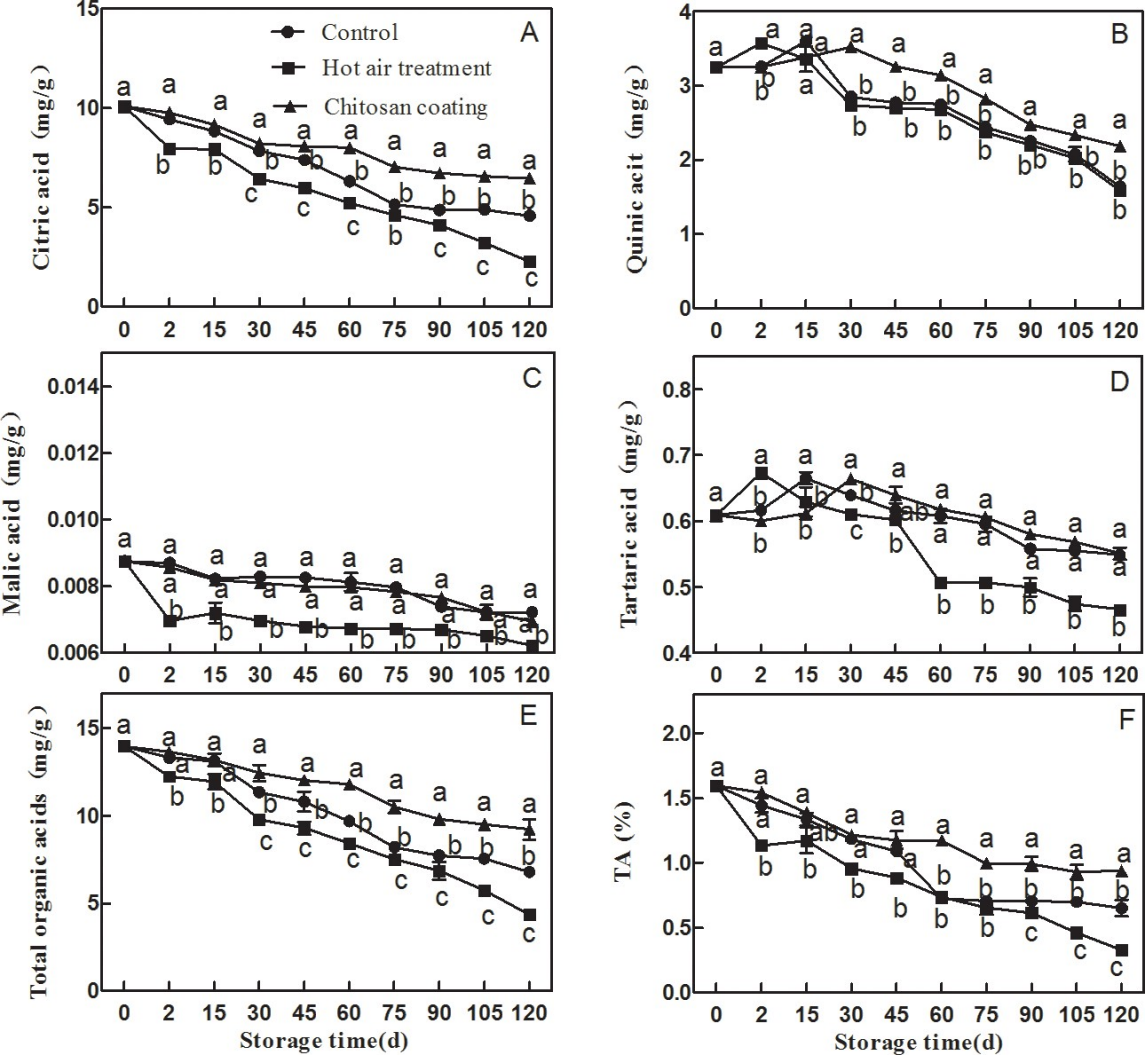
**Effect of hot air and chitosan coating on changes in the contents of citric (A), quinic (B), malic (C), tartaric (D), total organic acids (E) and TA (F) in flesh of Ponkan fruit during storage**. Vertical bars represent SD (n = 3). Significant differences (p <0.05) between means are indicated by different letters.

Organic acids, including citric acid, tartaric acid, malic acid and quinic acid, were measured during Ponkan fruit storage “Fig 1”. Citric acid, the major organic acid in Ponkan fruit, showed a decreasing trend during storage. Compared with the control, HAT significantly accelerated the decrease of citric acid content in Ponkan fruit, but chitosan coating significantly delay the decrease of citric acid. Quinic acid and tartaric acid content increased first and then decreased in Ponkan fruit during storage, chitosan coating delay the decrease of quinic acid content in Ponkan fruit, HTA accelerated the decrease of tartaric acid content. Throughout storage, malic acid content was less than 0.01mg/g in Ponkan fruit, which had little influence on fruit acidity in Ponkan fruit.

As the main of organic acid in Ponkan fruit, the degradation of citric acid leaded to the decreasing of total organic acids and TA in fruit during storage “Fig 1”. In parallel, the content of total organic acids and TA value were substantially lower in HAT fruit and higher in chitosan coating fruit than in control fruit.

### Effect of HAT and chitosan treatment on the expression of *CitAcos*

**Fig 2:**
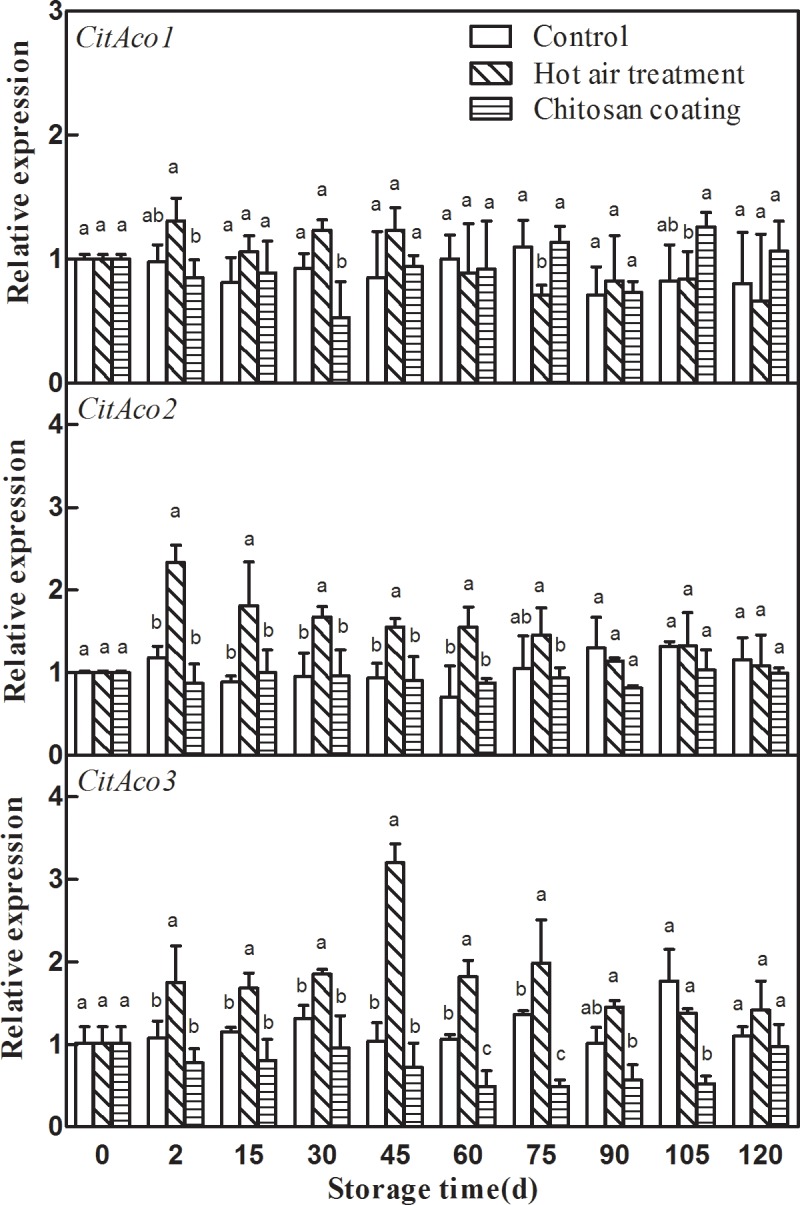
Effects of hot-air treatment and chitosan coating on expressions of *CitAcos* in Ponkan fruit. Vertical bars represent SD (n = 3). Significant differences (*p* <0.05) between means are indicated by different letters.

The effects of HAT and chitosan coating on expression patterns of three *CitAcos* genes were different. HAT and chitosan coating had no significant effect on the expression of *CitAco1*, and the relative expression level of *CitAco1* among the three treatments had no significant difference during the entire storage “Fig 2”. HAT transiently induced the expression of *CitAco2* and *CitAco3*, and maintained the transcript abundance till the 75 d of storage. The *CitAco2* expressions of HAT was 2.32 -fold of the initial value at 2d, the *CitAco3* expressions of HAT was 3.20 -fold of the initial value at 40d of storage “Fig 2”. However, chitosan coating decreased the expression of *CitAco2* and *CitAco3*. The stimulation of chitosan coating on CitAco2 expression was not significant, but the stimulation of chitosan coating on *CitAco3* expression was significant at 60d, 75d and 105d of storage “Fig 2”. Thus, the significant increase of *CitAco2/3* transcript levels, induced by HAT, may lead to the degradation of citric acid became more faster in HAT Ponkan fruit during storage, and the significant changes of *CitAco3* transcripts, inhibited by chitosan treatment, may lead to the retard degradation of citric acid in Ponkan fruit treated by chitosan. Comparing the relative expression levels of the three *CitAcos* genes, it was found that the relative expression level of *CitAco2* in HAT fruit at 2d was higher than that of *CitAco3* 2d and 15d of storage, and was lower at other storage time. The relative expression level of *CitAco2* in chitosan coating fruit was higher than that of *CitAco3*, and the difference was greater in the middle and late stages of storage. This indicated that *CitAco2* played a major role in the degradation of citric acid in the early storage stage of citrus fruits, while *CitAco3* played a major role in the middle and late stages.

### Effect of HAT and chitosan coating on the expression of *CitIDHs*

**Fig 3:**
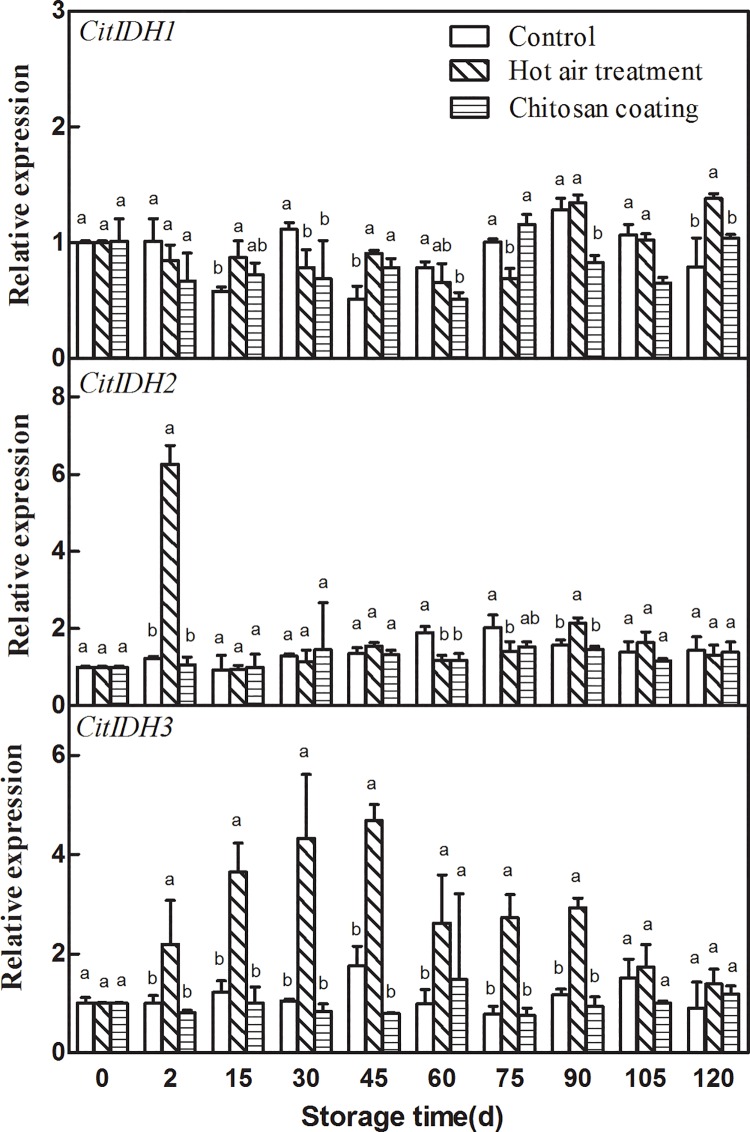
Effects of hot-air treatment and chitosan coating on expressions of *CitIDHs* in Ponkan fruit. Vertical bars represent SD (n = 3). Significant differences (*p* <0.05) between means are indicated by different letters.

As the similar response to the *CitAco* genes, the effects of HAT and chitosan coating on expression patterns of three *CitIDHs* genes were also different. HAT and chitosan coating had no significant effect on the expression of *CitIDH1*, but HAT transiently induced *CitIDH2* and *CitIDH3* expression, HAT transiently induced *CitIDH2* by 5.17-fold only at 2 d of storage “Fig 3”. HAT transiently induced *CitIDH3* by 2.19-fold at 2 d and maintain higher transcript level during the following storage, and peaked at 45 d of storage. On the contrast, chitosan coating decreased the expression level of *CitIDH2* and *CitIDH3*. The stimulations of chitosan coating on *CitIDH2* and *CitIDH3* expression were not significant.

### Effect of HAT and chitosan coating on the expression of *CitGADs* and *CitGS2*

**Fig 4:**
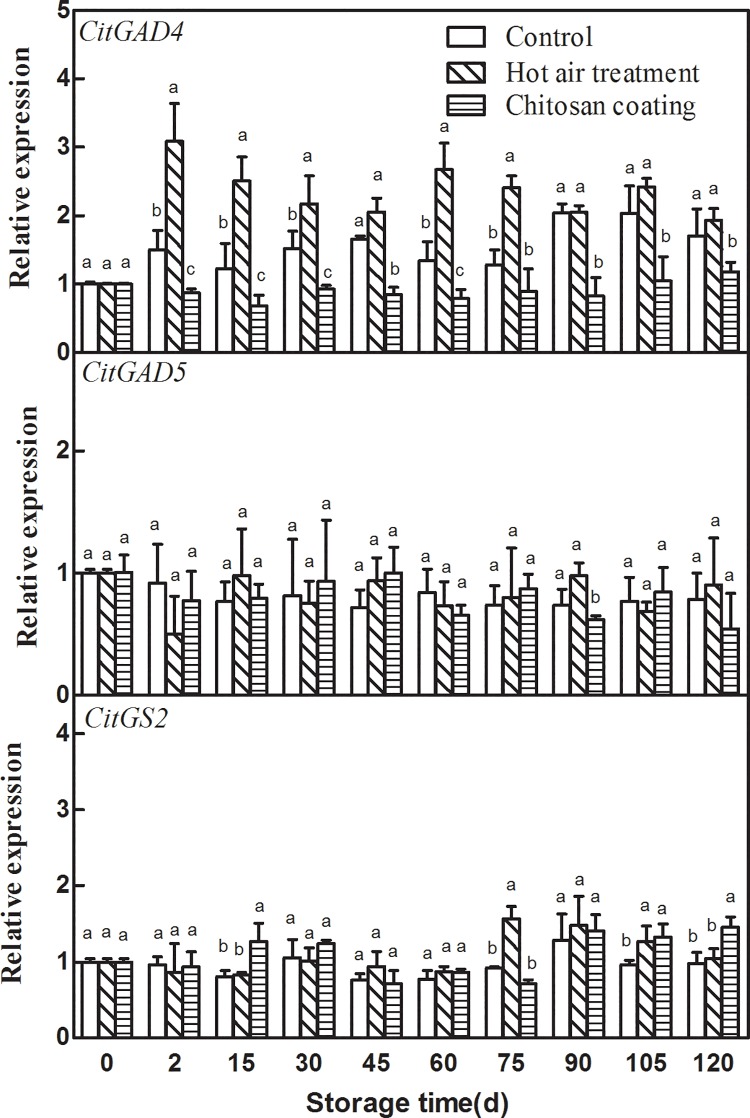
Effects of hot-air treatment and chitosan coating on expressions of *CitGADs* and *CitGS2* in Ponkan fruit. Vertical bars represent SD (n = 3). Significant differences (*p* <0.05) between means are indicated by different letters.

As presented in “Fig 4”, HAT and chitosan coating had significant effect on the expression of *CitGAD4*, but no significant effect on the expression of *CitGAD5* and *CitGS2*. The expression of *CitGAD4* was significantly higher in HAT fruit than control fruit during storage, which was 2.05 times of the control at 2 d. Chitosan coating significant decreased *CitGAD4* expression, which made *CitGAD4* expression in chitosan coating fruit significantly lower than the control.

### Effect of HAT and chitosan coating on the expression of *CitACLs*

**Fig 5:**
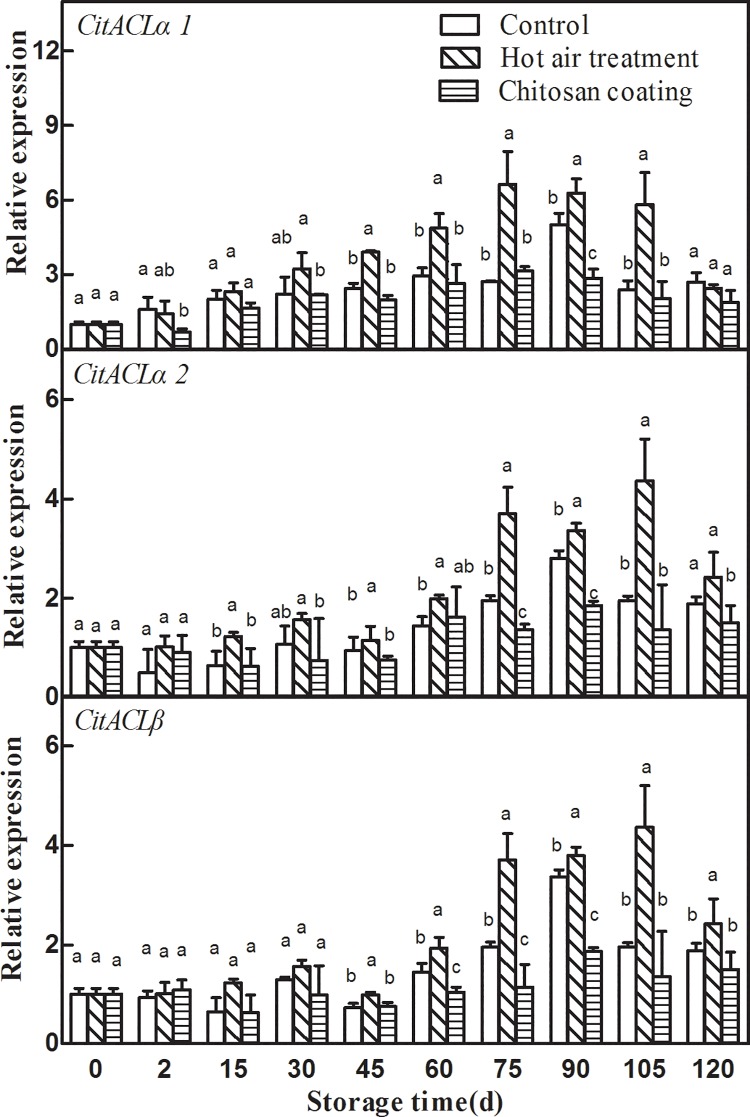
Effects of hot-air treatment and chitosan coating on expressions of *CitACLs* in Ponkan fruit. **Vertical bars represent SD (n = 3).** Significant differences (*p* <0.05) between means are indicated by different letters.

As presented in “Fig 5”, the effects of HAT and chitosan coating on expression patterns of three *CitACLs* genes were different. HAT significantly enhanced the expression of *CitACLs* family genes in Ponkan fruit during storage. The expression of *CitACLs* in HAT fruit increased at first and then decreased. The expression of *CitACLα1* reached the highest value at 75 d and *CitACLα2/β* were 105d. Chitosan coating decreased the expression level of *CitACLα2* and *CitACLβ*, which made *CitACLα2* and *CitACLβ* expression in chitosan coating fruit significantly lower than the control during storage.

### Effect of HAT and chitosan coating on the expression of *CitPEPCKs*

**Fig 6:**
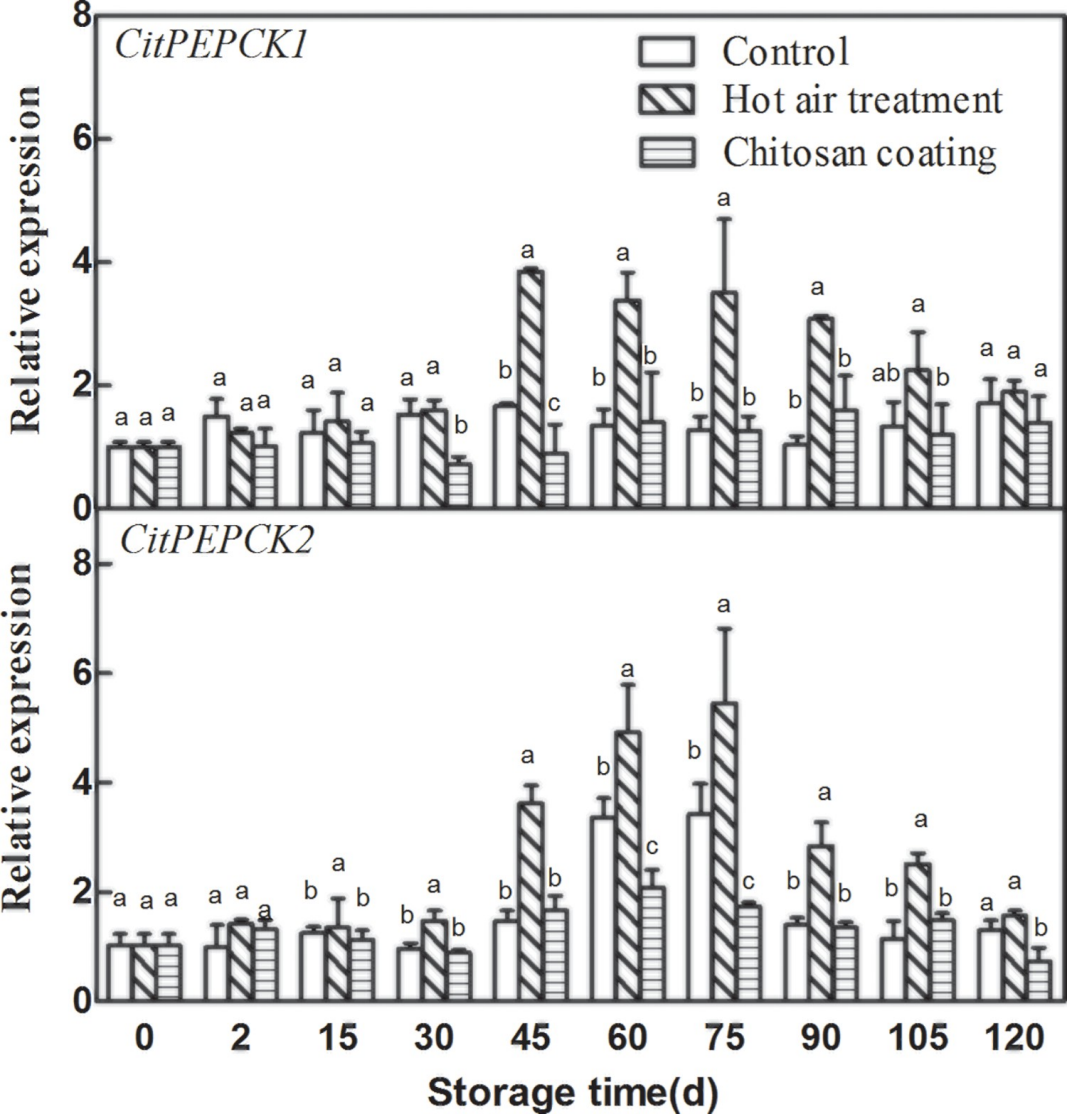
Effects of hot-air treatment and chitosan coating on expressions of *CitPEPCKs* in Ponkan fruit. **Vertical bars represent SD (n = 3).** Significant differences (*p* <0.05) between means are indicated by different letters.

The effects of HAT and chitosan coating on expression patterns of *CitPEPCKs* genes were different “Fig 6”. HAT induced the expression of *CitPEPCK1* and *CitPEPCK2*, and the expression of *CitPEPCKs* in HAT fruit was significantly higher than that of control. However, the expression of *CitPEPCK1* in chitosan coating fruit was significantly lower than that of control at 30 d and 45 d, and the expression of *CitPEPCK2* was 45 d and 60 d.

### Effect of HAT and chitosan coating on the expression of *CitFBPases*

**Fig 7:**
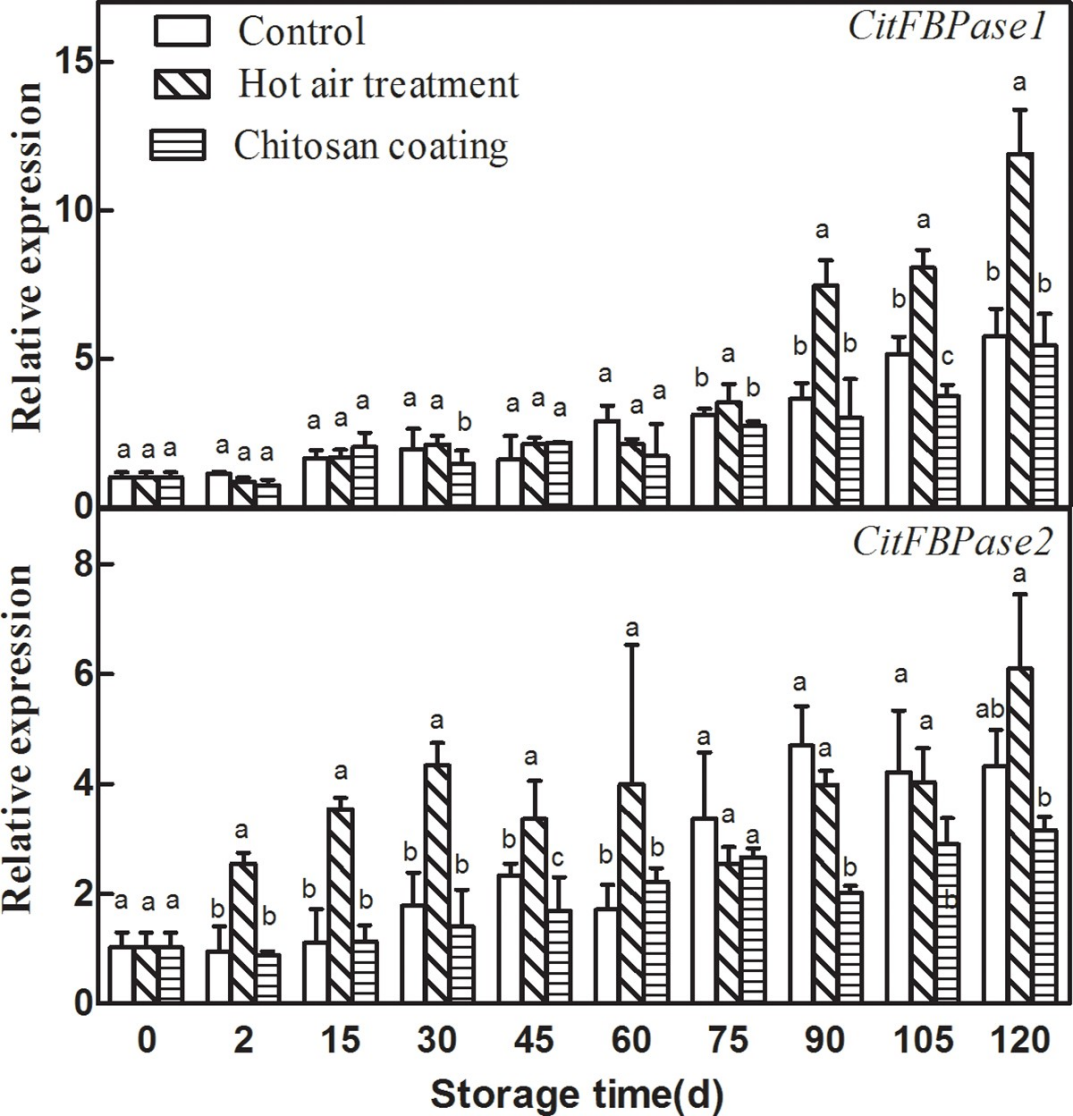
Effects of hot-air treatment and chitosan coating on expressions of *CitFBPases* in Ponkan fruit. **Vertical bars represent SD (n = 3).** Significant differences (*p* <0.05) between means are indicated by different letters.

As presented in “Fig 7”, the expression of two *CitFBPases* genes in Ponkan fruit increased gradually during storage. After treatment with HAT, the expression levels of two *CitFBPases* were significantly induced in Ponkan fruit, the expression of *CitFBPase1* was induced during the later storage and *CitFBPase2* was induced during the early storage. On the contrary, chitosan coating decreased *CitFBPases* expression. The expression of *CitFBPase2* in chitosan coating fruit was significantly lower than that of control during the later storage.

## Discussion

Citrus is a typical non-transgenic fruit, and its quality decreases gradually during post-harvest storage [27]. Many studies had been shown that the appropriate postharvest treatments could control the post-harvest storage quality of fruit. The soluble sugar metabolism was related to the cold resistance of peach fruit, and the sucrose content of peach fruit treated with hot air treatment and methyljasmonate was increased and the fruit’s tolerance to cold was enhanced [28]. The CMC/chitosan bilayer coating could effectively improve fruit firmness during citrus storage, especially on grapefruit [22].

A lots of study on organic acids metabolism in citrus fruit had been reported. Yamaki et al. [29] analyed the organic acids components in 47 citrus fruit juices. Citrus fruit contained citric acid, tartaric acid, quinic acid, malic acid, acetic acid, oxalic acid and so on. 44 citrus were citric acid-based fruit, which accounting for 75.4% -96.9% of organic acids content. The other 3 citrus were malic acid accumulation type, malic acid occupied 55.8% -60.1% of organic acid content. The results of HPLC showed that citric acid was the main organic acid in Jing’an Ponkan fruit, which was consistent with the results of Carballo et al. [30]. As shown in Fig 1, Hot air treatment could significantly promote the decline of citric acid, the content of citric acid and total organic acid in the fruit was significantly lower than that in the control [25,31]. On the contrast, chitosan coating could significantly delayed the degradation of citric acid and maintained high level of organic acid content in ponkan fruit at the later stage of storage.

The organic acid metabolism in citrus fruit is very complicated, which includes the synthesis, degradation and transportation of citric acid. Tadeo [32] proposed a citric acid synthesis pathway: phosphoenolpyruvate (PEP) immobilized CO2 to form oxaloacetic acid (OAA) catalyzed by phosphoenolpyruvate carboxylase (PEPC), and OAA combined with acetyl coenzyme A to produce citric acid under the catalysis of citric acid synthase (CS). The citric acid content of ‘Newhall’ navel orange [33] and Ponkan [34] fruits decreased gradually at the late stage of fruit development, which was not directly related to citric acid synthesis genes *CitCSs* and *CitPEPCKs* expression, it is mainly influenced by the genes related to degradation. Chen [25] suggested that there was no significant correlation between postharvest organic acid metabolism and synthetical genes, suggesting that the regulation of organic acids in citrus fruits was not related to citric acid biosynthesis.Citric acid could be degraded in different ways, including acetyl coenzyme A pathway (ACL) pathway, glutamine (GS) pathway and gamma aminobutyric acid (GABA) pathway. Citric acid could produce acetoacetic acid and acetyl coenzyme A under the action of ACL, and then turn to sugar ISO pathway or synthesize secondary metabolites such as flavonoids and fatty acids through the action of PEPCK and FBPase [35]. CERCÓS [36] and Katz [37] reported that ACL participates in citric acid accumulation during citrus fruit development and maturation. Citric acid was decomposed to isocitrate in the presence of aconitase (Aco), then metabolized into 2-oxoglutamate and glutamate under the action of isocitrate dehydrogenase (IDH) [36]. On one hand, glutamate generated glutamine by the role of glutamine synthetase (GS), on the other hand glutamate was catalysed into γ-aminobutyric acid (GABA) pathway. The Aco activity of citrus fruit was significantly decreased and the content of citric acid was significantly increased after surface coating [38]. *CitNAC62* and *CitWRKY1* could transactivate the promoter of CitAco3, then improv the expression of CitAco3 to control the degradation of citric acid in citrus fruit [39]. Zhang et al. [3] found that 40% soil water stress treatment could significantly inhibit the expression of *CitIDHs* in Citrus fruit, and citric acid content was significantly increased, which was 66.3% higher than that of the control. The expression of *CitGAD4* and *CitGAD5* in citrus fruit was inhibited under the stress of water stress, and the degradation of citric acid was blocked and the organic acid was accumulated, which indicated the important role of GABA cycle on citric acid degradation in citrus fruit. Exogenous GABA treatment could inhibit the expression of glutamic acid decarboxylase (GAD) and increase the citric acid content of fruit. At the same time, the content of amino acid and the activity of resistance enzyme could be improved, and the fruit rot was reduced [7].

**Fig 8:**
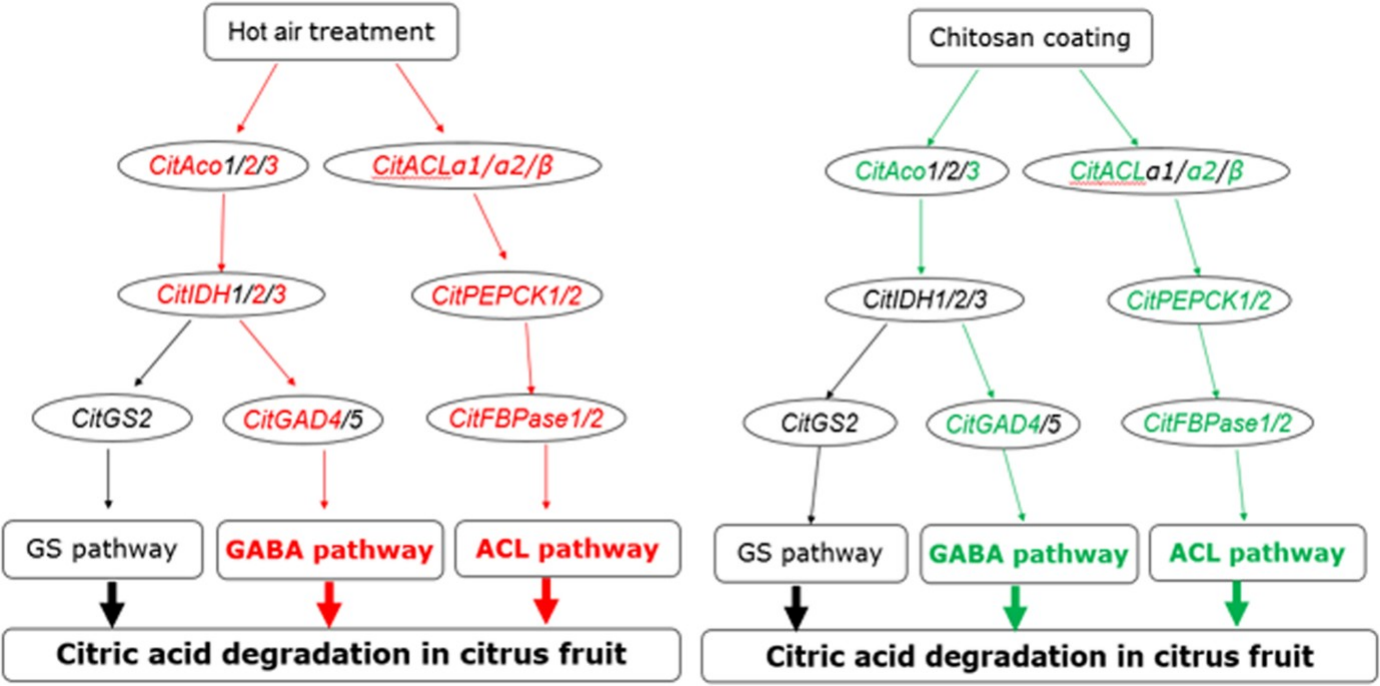
The model for degradation of citric in Ponkan fruit. Red indicates induction; Green indicates inhibition.

Our results showed that the expression of *CitAco2/3*, *CitIDH2/3*, *CitGAD4*, *CitACLs*, *CitPEPCKs* and *CitFBPases* could be induced by HAT, but HAT had no significant effect on the expression of *CitGSs*. The increased expression of these citrate degradation-related genes was associated with the degradation of citric acid, which led to the lower citric acid level in HAT Ponkan fruit than that of control during storage. Chitosan treatment could inhibit the expression of *CitAco3*, *CitGAD4*, *CitACLα2/β*, *CitPEPCKs* and *CitFBPases* during storage, and the degradation rate of citric acid in chitosan coating Ponkan fruit was slowed down during storage. These results suggested that citric acid may be degraded mainly through ACL pathway and GABA pathway during postharvest storage of Ponkan fruit “Fig 8”.

## Conclusions

Based on the present research, it was concluded that hot air treatment could induced the expression of *CitAco2/3*, *CitIDH2/3* and *CitGAD4* genes, which resulted in the acceleration of citric acid degradation through GABA pathway during Ponkan fruit storage. The chitosan coating inhibited the expression of *CitAco3* and *CitGAD4* genes, which caused the degradation of citric acid became slower in citrus fruit during storage. The specific regulation mechanism of citric acid metabolism in citrus fruit remains to be further studied in many ways, such as citric acid transport, and through the means of transcriptional group, metabolic group and proteomics.
